# Clinical and pathogen features of COVID-19-associated infections during an Omicron strain outbreak in Guangzhou, China

**DOI:** 10.1128/spectrum.03406-23

**Published:** 2024-09-06

**Authors:** Lin-ling Cheng, Zheng-tu Li, Hong-kai Wu, Feng Li, Ye Qiu, Tao Wang, Hui Peng, Zi-hao Liu:, Pan-rui Huang, Lu Zhou, Li-fen Gao, Hui-ju Huang, Bin Zhang, Xi-long Deng, Xin Chen, Feng Ye, Xiao-qing Liu, Wei-jie Guan, Yue-ping Li, Yi-min Li, Shi-yue Li, Nan-shan Zhong

**Affiliations:** 1 State Key Laboratory of Respiratory Disease, National Clinical Research Center for Respiratory Disease, Guangzhou Institute of Respiratory Health, The First Affiliated Hospital of Guangzhou Medical University, Guangzhou, China; 2 Guangzhou Eighth People`s Hospital, Guangzhou Medical University, Guangzhou, China; 3 Department of Pulmonary and Critical Care Medicine, Zhujiang Hospital, Southern Medical University, Guangzhou, China; National Chung Hsing University, Taichung, Taiwan, China

**Keywords:** Omicron strain, COVID-19, co-infection, superinfection, diabetes mellitus, *Klebsiella pneumoniae*, *Aspergillus*

## Abstract

Although the Omicron variant has been associated with greater transmissibility and tropism of the upper respiratory tract, the clinical and pathogenic features of patients infected with the Omicron variant during an outbreak in China have been unclear. Adults with COVID-19 were retrospectively enrolled from seven medical centers in Guangzhou, China, and clinical information and specimens ( BALF, sputum, and throat swabs) from participants were collected. Conventional detection methods, metagenomics next-generation sequencing (mNGS), and other methods were used to detect pathogens in lower respiratory tract samples. From December 2022 to January 2023, we enrolled 836 patients with COVID-19, among which 56.7% patients had severe/critical illness. About 91.4% of patients were infected with the Omicron strain (BA.5.2). The detection rate of possible co-infection pathogens was 53.4% by mNGS, including *Klebsiella pneumoniae* (16.3%), *Aspergillus fumigatus* (12.2%), and *Pseudomonas aeruginosa* (11.8%). The co-infection rate was 19.5%, with common pathogens being *Streptococcus pneumoniae* (11.5%), *Haemophilus influenzae* (9.2%), and *Adenovirus* (6.9%). The superinfection rate was 75.4%, with common pathogens such as *Klebsiella pneumoniae* (26.1%) and *Pseudomonas aeruginosa* (19.4%). *Klebsiella pneumoniae* (27.1%% vs 6.1%, *P* < 0.001), *Aspergillus fumigatus* (19.6% vs 5.3%, *P* = 0.001), *Acinetobacter baumannii* (18.7% vs 4.4%, *P* = 0.001), *Pseudomonas aeruginosa* (16.8% vs 7.0%, *P* = 0.024), *Staphylococcus aureus* (14.0% vs 5.3%, *P* = 0.027), and *Streptococcus pneumoniae* (0.9% vs 10.5%, *P* = 0.002) were more common in severe cases. Co-infection and superinfection of bacteria and fungi are common in patients with severe pneumonia associated with Omicron variant infection. Sequencing methods may aid in the diagnosis and differential diagnosis of pathogens.

**IMPORTANCE:**

Our study has analyzed the clinical characteristics and pathogen spectrum of the lower respiratory tract associated with co-infection or superinfection in Guangzhou during the outbreak of the Omicron strain, particularly after the relaxation of the epidemic prevention and control strategy in China. This study will likely prompt further research into the specific issue, which will benefit clinical practice.

## INTRODUCTION

During the global coronavirus pandemic, severe acute respiratory syndrome coronavirus 2 (SARS-CoV-2) evolved into multiple variant strains that are characterized by greater capacity of interpersonal transmission and decreased pathogenicity (1, 2). As of May 2023, there have been more than 766 million confirmed cases of coronavirus disease 2019 (COVID-19), including nearly seven million deaths globally (3).

Since January 2022, the Omicron variant has become the global dominant strain, with distinct clinical manifestations among different individuals. Although most patients had only developed self-limited upper airway symptoms (4), there remain some patients who may warrant hospitalization or admission to the intensive care unit. The Omicron strains have been frequently linked to more prominent upper airway symptoms, which differed considerably from the ancestor strains such as delta strain (4). Previous studies have reported that a considerable proportion of patients who were infected with COVID-19 had co-infection and/or superinfection in the lower respiratory tract (5, 6). Because SARS-CoV-2 may impair airway clearance against the invading pathogens, co-existing bacterial and fungal infections might be common (2, 7, 8).

Superinfection, especially COVID-19-associated fungal infections, could be life-threatening among some patients with COVID-19 (9). Co-infection and/or superinfection may markedly increase the risk of severe COVID-19 or mortality. Some studies have documented the preliminary findings of COVID-19-associated coinfection/superinfection with bacteria, viruses, and mycobacteria, as well as the clinical characteristics of the infected individuals (5, 6). Patients infected with multiple pathogens have greater disease severity than those infected with a single pathogen, as detected with conventional detection assays or metagenomics next-generation sequencing (mNGS) (2, 7, 8). However, the clinical characteristics and coinfection/superinfection of lower airways after the major outbreak of the Omicron strain in China have not been investigated.

In this study, we have retrospectively enrolled adult patients infected with Omicron strains from seven tertiary medical centers in Guangzhou, China. We employed multiple methods to detect pathogens, including conventional pathogen detection assays and mNGS, to investigate the clinical and pathogenic features of COVID-19-associated infections, with a specific focus on coinfections and superinfections, thus providing scientific evidence for enhancing the clinical management of COVID-19.

## MATERIALS AND METHODS

### Study population

This was a retrospective multicenter study that enrolled hospitalized patients with confirmed COVID-19 between 16 December 2022 and 19 January 2023 from seven tertiary medical centers in Guangzhou, China. The diagnosis, clinical classification, and disease severity of COVID-19 were made based on the COVID-19 Diagnosis and Treatment Plan (trial version 10), issued by the Ministry of Health, China (9) .

Eligible hospitalized patients were aged at least 18 years and had a confirmed diagnosis of COVID-19 [positive findings of rapid antigen test, or high-throughput metagenomic sequencing, or real-time reverse-transcriptase polymerase-chain-reaction (RT-PCR) assay of nasal and pharyngeal swab]. Key exclusion criteria were incomplete core clinical data (demographic information, comorbidities, laboratory results, or clinical outcomes), missing information regarding the detection status of microorganisms (including SARS-CoV-2), and other clinical conditions unsuitable for enrollment such as pregnancy, unqualified specimens, and patients who are unwilling to cooperate.

The Institutional Ethics Review Board of the First Affiliated Hospital of Guangzhou Medical University approved the study (No. 2020–92), in accordance with the guidelines of Good Clinical Practice and the Declaration of Helsinki. Written informed consent was waived in light of the urgent need to collect data during the upsurge of cases in the Omicron outbreak. All authors reviewed the manuscript and vouch for the accuracy and completeness of the data.

### Data sources

We entered clinical data (symptoms, signs, pathogen data, and imaging features) into the predefined information collection sheet, which was similar to the information sheet for collecting clinical characteristics of severe acute respiratory infections (the International Severe Acute Respiratory and Emerging Infection Consortium) (10). Clinical data were collected, entered, and verified by two independent researchers for cross-validation. Patients were classified according to their initial disease severity category: severe (severe or critical illness) and non-severe group (mild or moderate illness) (9).

### Collection, storage, and detection of clinical specimens

The main clinical specimens included bronchoalveolar lavage fluid (BALF), induced sputum, and throat swabs. The specimens were initially stored in sterile containers, followed by immediate transportation (not exceeding 24 hours if promptly stored in 4℃ freezers) to the laboratory for testing. Pathogens were detected with conventional detection assays in the laboratory of each participating sites and mNGS in the central laboratory. The determination of causative agents was based on a comprehensive assessment by two experienced physicians, considering multiple factors. These factors included the patients’ clinical symptoms, infection indicators such as elevated procalcitonin or C-reactive protein levels, and observed changes in these indicators after sensitive antibiotic treatment. Additionally, we referred to the background information provided by the mNGS platform developers, which helped in distinguishing potential background colonization from true infection. In cases of disagreement between the two physicians, a third physician will intervene. Specifically, we defined infections identified within the first 48 hours of hospitalization as coinfections, while those detected after 48 hours (2 days）of admission were classified as superinfections (11–14).

#### Conventional detection

Smearing of specimens for microorganism staining, pathogen culture, antigen detection (G/GM test, T-Spot, and cryptococcus capsular antigen detection), antibody detection, and molecular detection (PCR) were performed according to the methodology of each participating site.

#### mNGS

Specimens (only BALF samples) were initially stored in −80°C freezers. Mechanical wall-breaking treatment (NOVAprep DS1000 grinding instrument, 4,500 rpm/45 seconds/two cycles) was performed, followed by nucleic acid extraction, enzyme digestion, end repair, and adapter and unbiased PCR amplification for library construction (PMseq high-throughput kit, Z000100069). Next, the library was subject to single-strand nucleic acid cyclization. After rolling circle replication to generate DNB nanospheres, sequencing with a single-end read length of 50 bp was performed by using the MGISEQ-2000 gene sequencer. The sequencing data were processed using the local metagenomic server PMseq Datician pathogen expert analysis system. For sequencing data processing, the low-quality reads and reads less than 35 bp in length were initially removed, followed by removal of human reference genome sequence data by using Burrows–Wheeler aligner (BWA) (http://bio-bwa.sourceforge.net/). The remaining data were compared with the pathogenic microorganism database [10,989 species of bacteria (including 196 species of divergence bacterium, 159 mycoplasma/*Chlamydia*/*Rickettsia* species), 1,179 species of fungi, 5,050 viral species, and 282 parasite species] after removal of low-complexity reads to obtain the sequence number that could match a pathogen (the pathogenic microorganism database, http://ftp.ncbi.nlm.nih.gov/genomes/). Finally, the sequencing data were assigned to a taxonomic category for the viruses, bacteria, fungi, and parasites (10, 15).

In line with other published studies, co-infection was defined as clinically significant positive results from samples collected within 2 days of admission, while superinfection refers to the detection of one or more pathogenic species in a respiratory sample at least 2 days after hospitalization, which was synonymous with hospital onset or hospital-acquired infection.

#### SARS-CoV-2 variant detection

Nucleic acid was extracted by using nucleic acid extraction (RNA) or Purification (Sansure Biotech, S10015) on Sansure Purification System, and detected by the SARS-CoV-2 Omicron Delta RT-qPCR Detection Kit (Basogene,BR-III-108). The kit uses One-Step Real-time PCR and TaqMan fluorescent probe technology to target highly conserved regions and mutation sites of Omicron and Delta strains, including 6,970 del, KSF141-143-, G252V, V289I, R346K, R346T, 6970DEL, KSF141-143-, G252V, V289i, R346K, R346T, K444T, L452Q, L452R, F486P, T547K, P681R, P681H, T842I, G19, and T11A.

### Statistical analysis

R (version 4.2.1) was used to perform statistical analysis. Continuous variables were displayed with the median (25th and 75th quartile) and tested for with the Wilcoxon rank-sum test. Categorical variables were presented with the number and percentage of cases and tested by using the χ test or Fisher’s exact test. The difference was considered statistically significant when the two-sided *P* value was less than 0.05. The univariable and multivariable logistic regression analyses were performed by the function of glm in R. The regression model was built by the logical variables of underlying disease, inflammatory markers (IL-6, CRP, and PCT), and prior glucocorticoid treatment.

## RESULTS

### Baseline demographics and clinical characteristics

**Fig 1:**
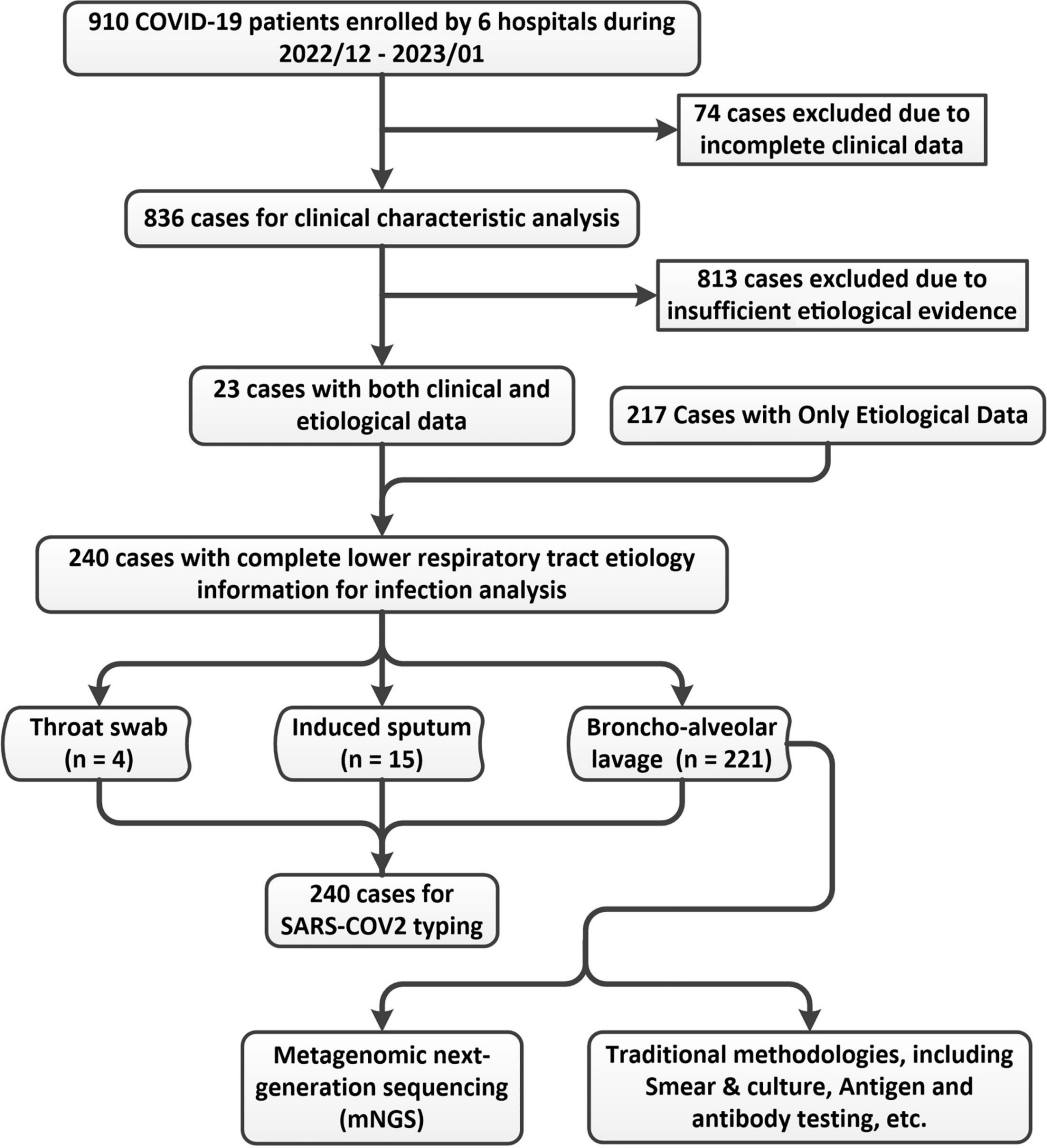
Analysis flow chart.

During the period of this study, 910 cases (COVID-19 hospitalized patients) were diagnosed between 16 December 2022 and 19 January 2023. After excluding 74 cases due to incomplete core clinical data, the remaining 836 cases were included to analyze the clinical characteristics. Furthermore, 23 cases from the 836 cases and other 217 cases from another study cohort, 240 cases in total, were enrolled with complete lower respiratory tract etiology information for infection analysis. Pathogens were detected from BALF by mNGS in 221 (221/240, 92.1%) patients (Fig. 1). At admission, 246 (29.4%) cases had critical illness, 228 (27.3%) had severe illness, 291 (34.8%) had moderate illness, and 71 (8.5%) developed mild illness only. Therefore, 56.7% of patients were categorized as severe and 43.3% were categorized as the non-severe group.

The median age was 75 years, with patients in the severe group being significantly older than those in the non-severe group (median: 77 vs 72 years, *P* < 0.001). The main symptoms were cough (83.4%), fever (57.4%), shortness of breath (47.8%), fatigue (32.1%), and chest tightness (17.2%). However, significantly more number of patients in the severe group than those in the non-severe group had fever (61.1% vs 53.3%, *P* = 0.031) and shortness of breath (52.5% vs 42.8%, *P* = 0.008). Unvaccinated patients were more likely to develop severe illness, with 57.3% in the severe group and 49.4% in the non-severe group (*P* = 0.03). In addition, significantly fewer patients had been vaccinated with three doses of SARS-CoV-2 vaccines in the severe group compared with the non-severe group (23% vs 34.5%, *P* < 0.001).. Patients in the severe group had a markedly higher rate of diabetes (32.3% vs 19.9%, *P* < 0.001), cardiovascular disease (60.7% vs 53.3%, *P* = 0.033), and chronic kidney disease than those in the non-severe group (13.3% vs 6.9%, p= 0.003) (Table 1).

**TABLE 1:** Demographic and basic clinical characteristics of patients with and without severe illness of COVID-19^*a*^

Parameters	Total (*N* = 836)	Severe group (*N* = 474)	Non-severe group (*N* = 362)	*P*-value
Demographic characteristics				
Age median (IQR), year	75 (64–84)	77 (67–85)	72 (61–82)	<0.001
Gender, males n (%)	522/836 (62.4)	319/474 (67.3)	203/362 (56.1)	0.001
BMI median (IQR)	22.90 (20.24–25.14)	22.77 (20.00–25.19)	23.03 (20.62–25.11)	0.402
Smoking n (%)	161/755 (21.3)	79/393 (20.1)	82/362 (22.7)	0.393
Non-vaccination n (%)	406/758 (53.6)	227/396 (57.3)	179/362 (49.4)	0.030
Received three doses of the COVID-19 vaccine n (%)	216/758 (28.5)	91/396 (23.0)	125/362 (34.5)	<0.001
Days between onset and hospitalization median (IQR), day	7 (3-11)	6 (3-10)	8 (4-13)	<0.001
Underlying diseases [n (%)]				
Diabetes	225/836 (26.9)	153/474 (32.3)	72/362 (19.9)	<0.001
Cardiovascular diseases	480/835 (57.5)	287/473 (60.7)	193/362 (53.3)	0.033
Chronic lung disease	178/782 (22.8)	88/420 (21.0)	90/362 (24.9)	0.194
Chronic kidney disease	81/782 (10.4)	56/420 (13.3)	25/362 (6.9)	0.003
Chronic liver disease	32/782 (4.1)	19/420 (4.5)	13/362 (3.6)	0.512
Immunosuppression	13/816 (1.6)	10/454 (2.2)	3/362 (0.8)	0.119
Tumors	64/781 (8.2)	36/419 (8.6)	28/362 (7.7)	0.663
Nervous system diseases	113/782 (14.5)	70/420 (16.7)	43/362 (11.9)	0.058
Other underlying disease	174/706 (24.6)	83/344 (24.1)	91/362 (25.1)	0.756
Any underlying disease	717/808 (88.7)	409/446 (91.7)	308/362 (85.1)	0.003
Clinical signs and symptoms [n (%)]				
Fever	433/755 (57.4)	240/393 (61.1)	193/362 (53.3)	0.031
Cough	627/752 (83.4)	319/390 (81.8)	308/362 (85.1)	0.226
Short breath	357/747 (47.8)	202/385 (52.5)	155/362 (42.8)	0.008
Fatigue	240/748 (32.1)	130/386 (33.7)	110/362 (30.4)	0.335
Chest distress	128/745 (17.2)	61/383 (15.9)	67/362 (18.5)	0.351
Nasal congestion	28/740 (3.8)	13/378 (3.4)	15/362 (4.1)	0.616
Runny nose	33/739 (4.5)	16/377 (4.2)	17/362 (4.7)	0.766
Muscle soreness	69/738 (9.3)	35/376 (9.3)	34/362 (9.4)	0.969
Hypogustatory	4/737 (0.5)	2/375 (0.5)	2/362 (0.6)	1.000
Palpitation	28/741 (3.8)	18/379 (4.7)	10/362 (2.8)	0.156
Diarrhea	21/836 (2.5)	12/474 (2.5)	9/362 (2.5)	0.967
Hemoptysis	21/737 (2.8)	6/375 (1.6)	15/362 (4.1)	0.038
Laboratory tests [median (IQR)]				
PH	7.406 (7.362–7.443)	7.410 (7.359–7.451)	7.402 (7.372–7.433)	0.346
PaO2 (mmHg)	86.6 (66.0–114.0)	77.3 (58.4–106.0)	96.6 (79.8–130.1)	<0.001
PaCO2 (mmHg)	36.0 (31.0–41.5)	35.2 (30.0–41.1)	37.1 (33.0–41.9)	0.003
HCO3- (mmol/L)	23.2 (20.7–25.5)	23.0 (20.3–25.5)	23.3 (21.4–25.5)	0.106
WBC (10^9^ /L)	7.29 (5.32–10.61)	8.04 (5.83–12.60)	6.36 (4.85–8.51)	<0.001
PLT (10^9^ /L)	203 (150–270)	192 (141–248)	226 (161–288)	<0.001
Lymphocyte count (10^9^ /L)	0.90 (0.56–1.40)	0.78 (0.50–1.24)	1.10 (0.72–1.62)	<0.001
Procalcitonin (μg/L)	0.09 (0.03–0.42)	0.21 (0.06–1.41)	0.04 (0.02–0.12)	<0.001
C-reactive protein (mg/L)	25.99 (8.56–89.58)	50.19 (14.00–126.78)	12.80 (3.39–34.99)	<0.001
Interleukin-6 (pg/mL)	16.52 (5.60–52.56)	34.66 (9.49–120.65)	10.73 (3.35–27.09)	<0.001
Interleukin-8 (μg/L)	46.15 (24.59–113.08)	47.03 (22.14–100.56)	44.46 (27.75–119.94)	0.572
Ferritin (ng/mL)	838 (407–1426)	962 (520–1711)	692 (341–1265)	0.041
ESR (mm/h)	50 (25–77)	55 (28–86)	45 (24–71)	0.034
Creatinine (mg/dl)	86.1 (71.0–113.8)	89.0 (71.0–123.2)	84.3 (71.0–100.5)	0.034
Urea nitrogen (mmol/L)	6.8 (4.8–10.6)	8.3 (5.5–13.9)	5.6 (4.2–7.8)	<0.001
Positive SARS-CoV-2 IgM n (%)	58/252 (23.0)	28/136 (20.6)	30/116 (25.9)	0.322
Positive SARS-CoV-2 IgG n (%)	207/306 (67.6)	87/152 (57.2)	120/154 (77.9)	<0.001

^*a*^ Data are expressed as the median ±interquartile range. Fisher’s exact test and the Kruskal–Wallis H test were used to determine statistical significance among the groups. *P* < 0.05. Data were collected under sterile conditions before the patient received antimicrobial therapy treatment and during the active stage of the infection.

### SARS-CoV-2 variants

To explore the clonal information of SARS-CoV-2, 240 specimens (including 202 BALF specimens, 31 induced sputum specimens, and seven throat swabs) were subject to SARS-CoV-2 detection and molecular typing. Of these, 54 specimens could not be typed due to the low viral loads. The Omicron strain dominated across all SARS-CoV-2 strains, with the main molecular subclones being BA.5.2 (91.4%). Only a minority of specimens tested positive to BF.7 (4.8%), BA.5.2.1 (1.6%), BA.5 (1.6%), and XBB (0.5%). No other strains circulating globally, such as BQ1, were identified.

### The prevalence of lower respiratory tract co-infection and/or superinfection

**Fig 2:**
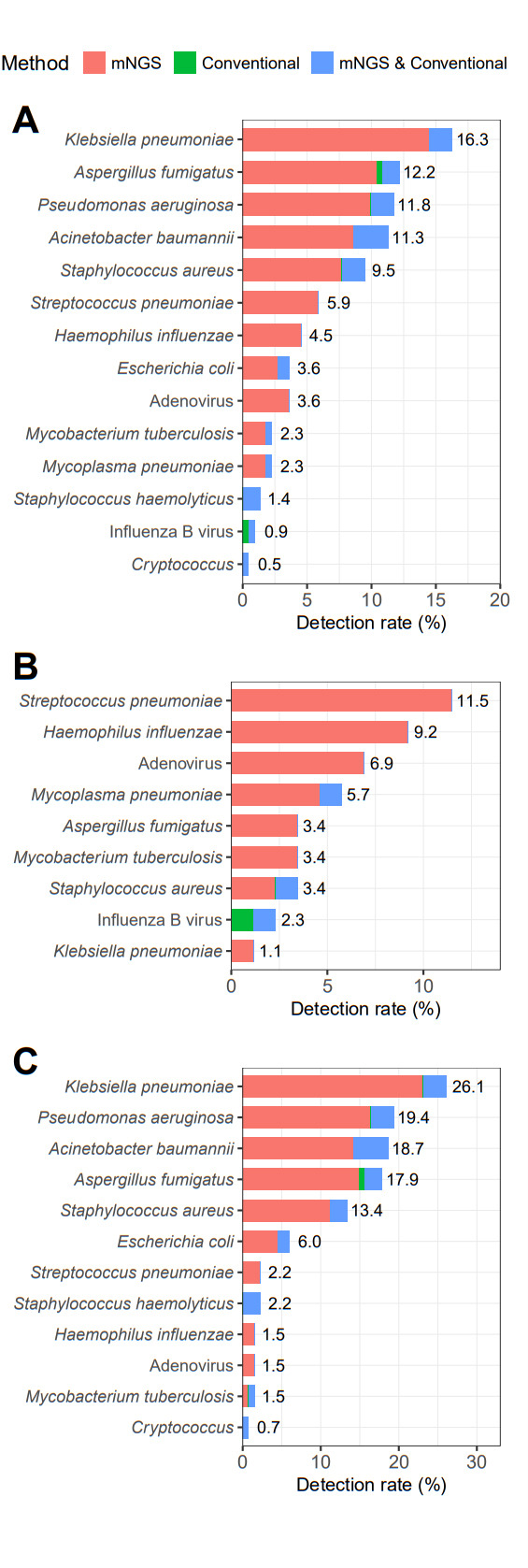
The pathogen spectrum of co-infection and/or superinfection in patients with COVID-19 during the Omicron outbreak. The pathogen spectrum in patients with COVID-19 detected by mNGS and conventional methods are shown in the bar graph. Red bars represent the proportion of pathogens detected by mNGS only, green bars represent the proportion of pathogens detected by conventional methods only, and blue bars represent the proportion of pathogens detected by both conventional methods and mNGS. (**A**) Pathogens of COVID-19-associated infections. (**B**) pathogen of Co-infection associated with COVID-19. (**C**) Pathogen of superinfection associated with COVID-19.

To investigate the co-infection and/or superinfection status in patients with COVID-19, mNGS of BALF from 221 patients was performed, along with conventional pathogenic assays (smear, culture, GeneXpert, G/GM test, and cryptococcal antigen). After eliminating the colonizing, contaminating, and background pathogen, the detection rate of the strictly defined pathogenic bacteria and conditional pathogenic bacteria based on clinical considerations (symptoms and infection indicators such as elevated procalcitonin or C-reactive protein levels and decreased levels after sensitive antibiotic treatment) could be calculated. Overall, the detection rate of potentially pathogenic pathogens was 53.4% (118/221), with a significantly lower detection rate of conventional pathogenic assays (14.5%, 32/221). The detection rates were as follows: *Klebsiella pneumoniae* (16.3%, 36/221), *Aspergillus fumigatus* (12.2%, 27/221), *Pseudomonas aeruginosa* (11.8%, 26/221), *Acinetobacter baumannii* (11.3%, 25/221), *Staphylococcus aureus* (9.5%, 21/221), *Streptococcus pneumoniae* (5.9%, 13/221), *Haemophilus influenzae* (4.5%, 10/221), *Adenovirus* (3.6%, 8/221), *Escherichia coli* (3.6%, 8/221), *Mycobacterium tuberculosis* (2.3%, 5/221), and *Mycoplasma pneumoniae* (2.3%, 5/221) (Fig. 2A).

BALF were collected from 87 patients and subject to pathogen detection within 2 days of admission via both mNGS and conventional pathogen assays. Of these, 17 identified one or more pathogens, with the co-infection rate being 19.5% (17/87). However, the detection rate of conventional pathogen assays was 2.3% only. The detection rates were as follows: *Streptococcus pneumoniae* (11.5%, 10/87), *Haemophilus influenzae* (9.2%, 8/87), *Adenovirus* (6.9%, 6/87), *Mycoplasma pneumoniae* (5.7%, 5/87), *Staphylococcus aureus* (3.4%, 3/87), and Influenza B virus (2.3%, 2/87) (Fig. 2B).

To determine the superinfection status, BALF was collected from 134 patients and subject to pathogen detection at 2 days after admission via mNGS and conventional pathogens assays. A total of 101 patients tested positive to one or more pathogens, with the rate of superinfection being 75.4% (101/134). However, the detection rate of conventional pathogen assays was 26.9% (36/134) only. The detection rates were as follows: *Klebsiella pneumoniae* (26.1%, 35/134), *Pseudomonas aeruginosa* (19.4%, 26/134), *Acinetobacter baumannii* (18.7%, 25/134), *Aspergillus fumigatus* (17.9%, 24/134), *Staphylococcus aureus* (13.4%, 18/134), and *Escherichia coli* (6%, 8/134) (Fig. 2C).

### Pathogen spectrum in BALF during the epidemic of the Omicron strain

**Fig 3:**
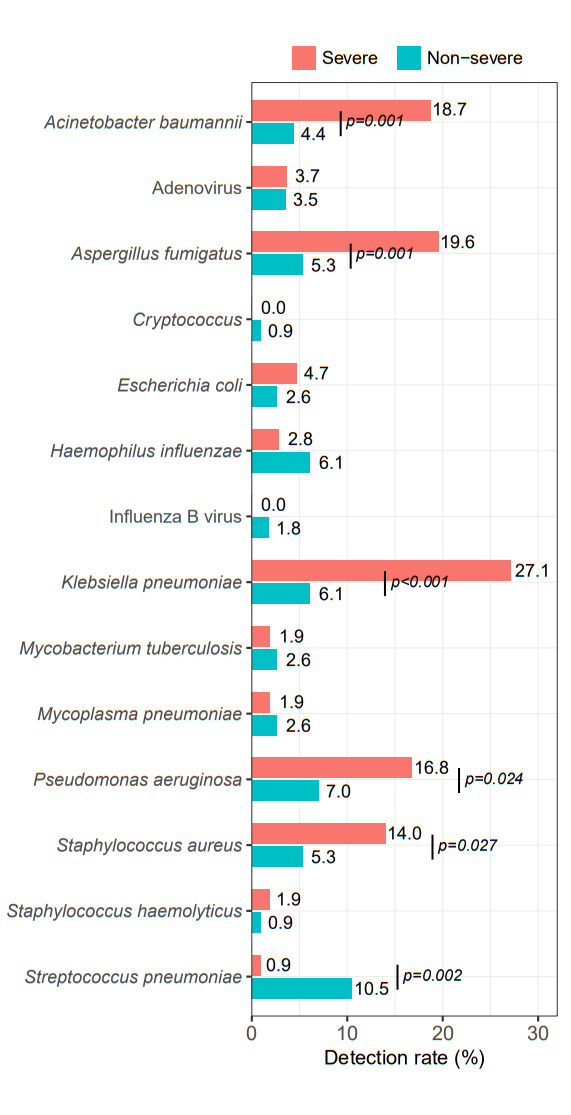
The pathogen spectrum of COVID-19-associated infections between the severe and non-severe groups. Bar graph showing the detection rate (%) of different pathogens in the severe and non-severe groups. The colors red and blue represent the severe and non-severe groups, respectively. The pathogens with the highest detection rates in the severe group included *Klebsiella pneumoniae*, *Acinetobacter baumannii*, and *Aspergillus fumigatus*. The pathogens with the highest detection rates in the non-severe group consisted of *Streptococcus pneumoniae, Pseudomonas aeruginosa, Haemophilus influenzae,* and *Klebsiella pneumoniae*. In addition, *Klebsiella pneumoniae, Aspergillus fumigatus, Acinetobacter baumannii,* and *Pseudomonas aeruginosa* were more common in the severe group than in the non-severe group.

In this study, BALF was obtained from 221 COVID-19 hospitalized patients by bronchoscopy. To evaluate the clinical characteristics and impact of COVID-19-associated infections to severe COVID-19 patients, we compared the pathogen spectrum in the severe (50.2%, 111/221) and non-severe groups (49.8%, 110/221). In the severe group, other common pathogens (apart from SARS-CoV-2) included *Klebsiella pneumoniae* (27.1%)*, Acinetobacter baumannii* (18.7%), *Aspergillus fumigatus* (19.6%)*, Pseudomonas aeruginosa* (16.8%), and *Staphylococcus aureus* (14.0%). The pathogens in the non-severe group were ordered as *Streptococcus pneumoniae* (10.5%), *Pseudomonas aeruginosa* (7.0%), *Haemophilus influenzae* (6.1%), *Klebsiella pneumoniae* (6.1%), *Aspergillus fumigatus* (5.3%), *Staphylococcus aureus* (5.3%), *Acinetobacter baumannii* (4.4%), *Adenovirus* (3.5%), *Mycoplasma pneumoniae* (2.6%), and Influenza B virus (1.8%). Furthermore, *Klebsiella pneumoniae* (27.1% vs 6.1%, *P* < 0.001), *Aspergillus fumigatus* (19.6% vs 5.3%, *P* = 0.001), *Acinetobacter baumannii* (18.7% vs 4.4%, *P* = 0.001), *Pseudomonas aeruginosa* (16.8% vs 7.0%, *P* = 0.024), *Staphylococcus aureus* (14.0% vs 5.3%, *P* = 0.027), and *Streptococcus pneumoniae* (0.9% vs 10.5%, *P* = 0.002) were more common in the severe group than in the non-severe group (Fig. 3).

Because of the high percentage of patients with superinfection with *Aspergillus* spp., we next analyzed the clinical characteristics of patients infected with *Aspergillus fumigatus*. COVID-19 superinfection with *Aspergillus fumigatus* were more common in patients with severe COVID-19, comorbid chronic airway diseases, low CD4^+^T cell count, and the receipt of medium-flow oxygen therapy (Table S1).

### Risk factors of co-infection and superinfection

**Fig 4:**
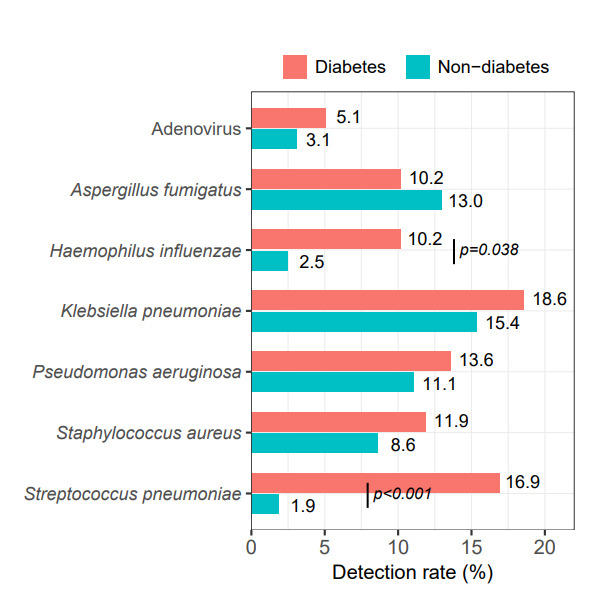
The influence of underlying diabetes on the pathogen spectrum in patients with COVID-19. A bar graph compares the detection rate of various pathogens in COVID-19 patients with and without diabetes. Red represents patients with diabetes, while green represents those without. Patients with diabetes had a higher detection rate of *Streptococcus pneumoniae*.

The associations between the co-infection and the clinical characteristics (e.g., underlying disease, inflammatory markers, immunity status, prior corticosteroid treatment, and COVID-19 vaccination) are shown in Table 2. Multivariable logistic regression analysis was performed to determine the factors associated with the co-infection. Diabetes (OR 43.16, 95% CI 8.20–358.10; *P* < 0.001) was independently associated with co-infection (Table 2). Further analysis revealed that COVID-19 patients with diabetes had a higher rate of pneumococcal infection (16.9% vs 1.9%, *P* < 0.001) and *Haemophilus influenzae* infection (10.2% vs 2.5%, *P* = 0.038) than those without diabetes (Fig. 4). There was no significant modifying effect of vaccination on the detection of pathogenic bacteria in COVID-19 patients (Table S2).

**TABLE 2:** Clinical factors associated with coinfection of COVID-19

Clinical factors	Coinfection of COVID-19		Univariable model^*a*^		Multivariable model^*b*^	
	No (*n* = 58)	Yes (*n* = 29)	OR (95% CI)	*P* value	OR (95% CI)	*P* value
Age median (IQR), y	74 (67–79)	73 (64–77)	1.01 (0.98–1.04)	0.723	−	−
Gender, males [No. (%)]	31/58 (53.4)	8/29 (27.6)	0.33 (0.12–0.85)	0.025	0.82 (0.16–4.67)	0.815
Smoking [No. (%)]	13/58 (22.4)	2/29 (6.9)	0.26 (0.04–1.02)	0.088	0.96 (0.09–7.38)	0.973
Prior glucocorticoid treatment [No. (%)]	29/58 (50.0)	14/29 (48.3)	0.93 (0.38–2.28)	0.879		
Underlying diseases [No. (%)]						
Diabetes	3/58 (5.2)	22/29 (75.9)	57.62 (15.50–294.37)	<0.001	43.16 (8.20–358.10)	<0.001
Cardiovascular disease	11/58 (19.0)	8/29 (27.6)	1.63 (0.56–4.63)	0.361	−	−
Chronic lung disease	11/58 (19.0)	8/29 (27.6)	1.63 (0.56–4.63)	0.361	−	−
Chronic kidney disease	6/58 (10.3)	5/29 (17.2)	1.81 (0.48–6.58)	0.366	−	−
Immunocompromised condition	7/58 (12.1)	1/29 (3.4)	0.26 (0.01–1.57)	0.219	−	−
Nervous system diseases	6/58 (10.3)	2/29 (6.9)	0.64 (0.09–3.01)	0.602		
Laboratory tests [median (IQR)]						
WBC count (10^9^ /L)	6.73 (4.87–9.51)	7.85 (5.67–10.83)	1.06 (0.96–1.19)	0.246	−	−
Lymphocyte count (10^9^ /L)	0.94 (0.70–1.60)	0.72 (0.51–1.58)	0.93 (0.76–1.05)	0.343	−	−
D-dimer (mg/L)	872 (509–1293)	999 (533–2024)	1.00 (1.00–1.00)	0.453	−	−
IL-6 (ng/L)	13.05 (6.15–36.59)	27.43 (12.82–45.55)	1.01 (0.99–1.02)	0.347	−	−
CRP (mg/L)	7.21 (2.25–43.51)	6.82 (4.59–145.63)	1.01 (1.00–1.02)	0.069	1.00 (0.99–1.01)	0.838
PCT (ng/mL)	0.04 (0.02–0.21)	0.28 (0.06–0.60)	1.01 (0.88–1.13)	0.874	−	−
COVID-19 vaccination [No. (%)]						
No vaccination	23/58 (39.7)	12/29 (41.4)	1.07 (0.43–2.66)	0.877	−	−
One or two dose	11/58 (19.0)	5/29 (17.2)	0.89 (0.26–2.75)	0.845	−	−
Three doses	24/58 (41.4)	12/29 (41.4)	1.00 (0.40–2.47)	1.000	−	−

^*a*^ Univariate logistic regression model was used to determine the associations between the coinfection and the clinical variables, including age, gender, smoking status, underlying diseases, inflammatory markers, immunity status, prior glucocorticoid treatment, and COVID-19 vaccination.

^*b*^ We only included the parameters with a *P* value of less than 0.1 in the univariable model into the multivariate logistic regression model: gender, smoking, diabetes, and CRP.

## DISCUSSION

Our study has analyzed the clinical and pathogenic features of COVID-19-associated infections, with a specific focus on coinfections and superinfections in Guangzhou during the outbreak of the Omicron strain, particularly after the relaxation of the epidemic prevention and control strategies in China. In this study, a high rate (88.7%) of comorbid underlying diseases was observed as compared with the findings from a published study (16). The rate of co-infection or superinfection, defined according to the conventional detection assays, was significantly lower than that detected with mNGS. *Klebsiella pneumoniae*, *Aspergillus fumigatus, Pseudomonas aeruginosa,* and *Acinetobacter baumannii,* which were normally hospital-acquired pneumonia-associated pathogens, were the most common pathogens associated with superinfection. By comparison, *Streptococcus pneumoniae*, *Haemophilus influenzae*, *Adenovirus,* and *Mycoplasma pneumoniae,* which were typically the community-acquired pneumonia-associated pathogens, were the most common pathogens associated with co-infection. *Klebsiella pneumoniae*, *Aspergillus fumigatus, Acinetobacter baumannii,* and *Pseudomonas aeruginosa* were common in severe cases than in non-severe cases.

The prevalent SARS-CoV-2 strain in Guangzhou during the study period was Omicron BA.5.2 (91.4%), which had less virulence and greater immune escape capacity compared with the ancestral wild-type strain. Previous studies have indicated the upper airway tropism of Omicron strains, which could in part explain the relatively low incidence of lower airway infection (17–19). However, these studies have suggested the likelihood of progression to severe disease, especially in patients with underlying disease, despite the overall mild disease severity at baseline. In our study, most hospitalized COVID-19 patients had developed lower respiratory tract infection and 56.7% developed severe illnesses. Advanced age, more underlying diseases, and low vaccination rate were associated with severe disease. Therefore, prompt identification of these risk factors would help rapid triage of the at-risk patients in clinical practice.

The evidence of COVID-19-related respiratory tract infections has been rapidly accumulating in other countries or regions (10, 15, 20, 21). For instance, the prevalence of COVID-19-associated airway infections varied between 3.2% and 3.6%.^10^, ^15^, ^20^, ^21^ However, in our study, the prevalence of COVID-19-associated airway infections reached 14.5% according to the conventional detection assays alone and 53.4% based on mNGS. The high detection rate of secondary infection or co-infection of COVID-19 in our study might have resulted from several factors, including the highly sensitive detection methods (high-throughput sequencing methods), the collection of lower respiratory tract samples BALF (higher sensitivity for detecting infection), and the sampling of patients in the intensive care unit (who have a higher risk of having secondary infection). Our study may have indicated the underdiagnosis of COVID-19-associated airway infections in clinical practice, including patients infected with the less virulent SARS-CoV-2 strains such as the Omicron BA.5.2 strain. Most hospitalized COVID-19 patients had at least one risk factor for community or nosocomial infections, such as older age and more underlying diseases, which might have resulted in the high rate of co-infection. Also, our study suggested that the high detection rate of co-infection pathogens in the lower respiratory tract of COVID-19 patients might be related to the patient’s underlying condition, including advanced age, co-existing diabetes, and the lack of receipt of vaccination. Besides, the reasons for the low incidence of COVID-19-associated infections in previous studies might be related to several key aspects. First, there remains a lack of a consensus regarding the operational case definition for COVID-19-associated infections. The invasive fungal disease case definition of the European Organization for Research and Treatment of Cancer/Mycosis Study Group Education and Research Consortium was rarely applicable because it could only be applied to patients with specific host factors, which were typically absent in patients suspected of having COVID-19-associated pulmonary aspergillosis (22). Second, it has been challenging to perform bronchoscopy to sample lower respiratory tract specimens during the first wave of the COVID-19 pandemic. The etiologic diagnosis mostly relied on the detection of sputum samples or throat swabs rather than BALF with conventional detection assays (bacterial and fungal culture) only, which might have yielded lower sensitivity for detecting secondary infections. Although bronchoscopy was not endorsed during the first wave of the COVID-19 outbreak, a careful evaluation for co-infection status could be indicated in some circumstances (23). Some studies have recently reported that bronchoscopy could be safely performed in COVID-19 patients (24), provided that adequate preventive measures were taken to protect healthcare workers (25). In our study, all pathogen assays were based on qualified lower respiratory tract specimens and BALF, obtained via bronchoscopy. Conventional pathogen detection assays such as bacterial and fungal culture and the emerging high-sensitivity detection methods such as mNGS have been applied for in-depth detection of the pathogens. These could have contributed to the markedly higher positive detection rate of other pathogens in the lower respiratory tract of COVID-19 patients.

To fully ascertain the detection of the true pathogens with NGS, we have only included the list of definite pathogens and the conditional pathogens by taking into account the clinical manifestations. Furthermore, *Klebsiella pneumoniae*, *Aspergillus fumigatus, Acinetobacter baumannii, Pseudomonas aeruginosa,* and *Staphylococcus aureus* were the most common pathogens associated with severe COVID-19. Moreover, the detection rate of *Mycobacterium tuberculosis* and influenza B virus was notable and should not be overlooked.

This study also had some limitations. First, our patients were mainly recruited from Guangzhou city, where the pathogenic spectrum might differ considerably from other regions due to environmental and patient underlying factors. Second, the cross-sectional study design did not allow for a comprehensive follow-up, and therefore we cannot comment on the impact of co-infection/superinfection on the prognosis. Furthermore, the definitions of co-infection and superinfection were not clear, although we have made a preliminary definition according to the important earlier published studies. Third, our study only recruited adult patients who were hospitalized, and therefore the results might not be representative of all patients with COVID-19 (particularly those who could be managed at). Additionally, it is worth noting the challenges in accurately distinguishing active infection from colonization when using highly sensitive molecular assays such as mNGS. Although mNGS can detect a broad range of microorganisms, including potential pathogens, it may also identify commensal or colonizing microorganisms that may not have clinical significance. Therefore, the interpretation of mNGS results should be approached cautiously, considering the potential for false-positive results or the identification of microorganisms not causally linked to the infection.

### Conclusion

Co-infection/superinfection of bacteria and fungi is common in patients with severe pneumonia following Omicron variant infection. Emerging diagnostic methods such as mNGS may also be helpful for the diagnosis and differential diagnosis of pathogens.

## Data Availability

Because the original sequencing data contained human genetic information, they were not available. However, a complete table of corresponding clinical data and mNGS sequencing data is attached for supplementary material (Tables S2 and S3).

## SUPPLEMENTAL MATERIAL

Table S1 spectrum.03406-23-s0001.docx The baseline clinical characteristics of patients with COVID-19 superinfected with aspergillus.

Table S2 spectrum.03406-23-s0002.xlsx The clinical information for 836 patients.

Table S3 spectrum.03406-23-s0003.xlsx Detection results of conventional method in BALF samples for 221 patients (raw data).

ASM does not own the copyrights to Supplemental Material that may be linked to, or accessed through, an article. The authors have granted ASM a non-exclusive, world-wide license to publish the Supplemental Material files. Please contact the corresponding author directly for reuse.
