# Supplementary material for: Clinical and pathogen features of COVID-19-associated infections during an Omicron strain outbreak in Guangzhou, China
Source: Microbiol Spectr. 2024 Sep 6;12(10):e03406-23. doi: 10.1128/spectrum.03406-23 (PMC11448415; doi:10.1128/spectrum.03406-23)
Supplement: Table S1 — The baseline clinical characteristics of patients with COVID-19 superinfected with aspergillus. [file spectrum.03406-23-s0001.docx]

**Supplementary Table 1. The baseline clinical characteristics of patients with COVID-19 superinfected with aspergillus**

| **Parameters** | **Total (N = 221)** | **Patients with Aspergillus infection (n=27)** | **Patients without Aspergillus infection (n=194)** | **P-value** |
| --- | --- | --- | --- | --- |
| **Demographic characteristics** |  |  |  |  |
| Age median (IQR)-years | 74 (62-80) | 75 (57-83) | 74 (62-80) | 0.891 |
| Gender-males n (%) | 103/221 (46.6) | 9/27 (33.3) | 94/194 (48.5) | 0.140 |
| Smoking n (%) | 56/221 (25.3) | 3/27 (11.1) | 53/194 (27.3) | 0.070 |
| No vaccination n (%) | 94/221 (42.5) | 12/27 (44.4) | 82/194 (42.3) | 0.830 |
| Received 3 dose of the COVID-19 vaccine n (%) | 86/221 (38.9) | 9/27 (33.3) | 77/194 (39.7) | 0.526 |
| Clinical typing-severe n (%) | 107/221 (48.4) | 21/27 (77.8) | 86/194 (44.3) | 0.001 |
| Prior glucocorticoid treatment n (%) | 96/221 (43.4) | 7/27 (25.9) | 89/194 (45.9) | 0.050 |
| **Basic diseases [n (%)]** |  |  |  |  |
| Diabetes | 59/221 (26.7) | 6/27 (22.2) | 53/194 (27.3) | 0.575 |
| Cardiovascular diseases | 45/221 (20.4) | 4/27 (14.8) | 41/194 (21.1) | 0.445 |
| Chronic lung disease | 50/221 (22.6) | 10/27 (37.0) | 40/194 (20.6) | 0.056 |
| Chronic kidney disease | 24/221 (10.9) | 2/27 (7.4) | 22/194 (11.3) | 0.775 |
| Immunosuppression | 19/221 (8.6) | 2/27 (7.4) | 17/194 (8.8) | 1.000 |
| Tumor | 11/221 (5.0) | 2/27 (7.4) | 9/194 (4.6) | 0.883 |
| Neurological disorder | 23/221 (10.4) | 4/27 (14.8) | 19/194 (9.8) | 0.643 |
| **Laboratory Tests [median (IQR)]** |  |  |  |  |
| WBC count(10^9^/L) | 7.19 (5.15-10.31) | 6.48 (4.85-7.37) | 7.37 (5.23-10.55) | 0.088 |
| Lymphocyte count (10^9^/L) | 0.90 (0.60-1.53) | 0.90 (0.59-1.65) | 0.90 (0.60-1.50) | 0.937 |
| D-dimer (mg/L) | 924 (523-1445) | 1074 (802-1585) | 902 (500-1428) | 0.147 |
| IL-6 (ng/L) | 15.52 (5.91-45.68) | 12.58 (6.42-38.69) | 15.98 (5.56-46.11) | 0.877 |
| CRP (mg/L) | 12.95 (3.54-59.48) | 17.99 (8.80-53.76) | 10.96 (3.47-59.48) | 0.574 |
| PCT (ng/ml) | 0.05 (0.03-0.27) | 0.06 (0.03-0.29) | 0.05 (0.02-0.25) | 0.486 |

Data are expressed as the median ± interquartile range. Fisher’s exact test and the Kruskal–Wallis H test were used to determine statistical significance among the groups. P < 0.05. Data were collected under sterile conditions before the patient received antimicrobial therapy treatment and during the active stage of the infection. HFNC: High-Flow Nasal Cannula.
